# Bleomycin electrosclerotherapy for kaposiform hemangioendothelioma with Kasabach–Merritt phenomenon in an adult

**DOI:** 10.1111/ddg.15951

**Published:** 2025-11-19

**Authors:** Jakob Veeser, Raphael Wilhelm, Janina Dietzel, Yalda Ghoreishi, Nora Laubach, Nora Elspaß, Maike Kaufhold, Beate Weidenthaler‐Barth, Christian Rose, Stephan Grabbe, Henner Stege, Hadrian Nassabi

**Affiliations:** ^1^ Department of Dermatology University Medical Center of the Johannes Gutenberg University Mainz Germany; ^2^ Dermato‐histological laboratory Bartsch und Rose Lübeck Germany

Dear Editors,

Kaposiform Hemangioendothelioma (KHE) is a rare tumor, which primarily manifests in childhood and adolescence.^1^ Nevertheless, rare cases in adulthood have also been reported.^2^, ^3^ KHE is often associated with Kasabach–Merritt phenomenon (KMP), a severe consumptive coagulopathy that can lead to disseminated intravascular coagulation (DIC) and increased bleeding risk.^4^ This report details a case of KHE with KMP in an adult patient and highlights the clinical and histological presentation, the diagnostic challenges and treatment strategies for this rare condition.

A 33‐year‐old female patient of Filipino descent presented with persistent bleeding from an exophytic, ulcerated tumor on her right upper arm, which had developed progressively over the past year. The patient reported a diagnosis of angiosarcoma of the left breast with consecutive mastectomy in her home country the previous year. There were no other pre‐existing conditions, and the family history was unremarkable. Clinically, there was an extensively bleeding tumor on the right upper arm, as well as multiple subcutaneous vascularized nodules on the trunk and upper extremities. (Figure 1a, b) The patient was in a good state of health and with an obese nutritional status. There was no unilateral overgrowth of an extremity or gigantism.

**FIGURE 1:**
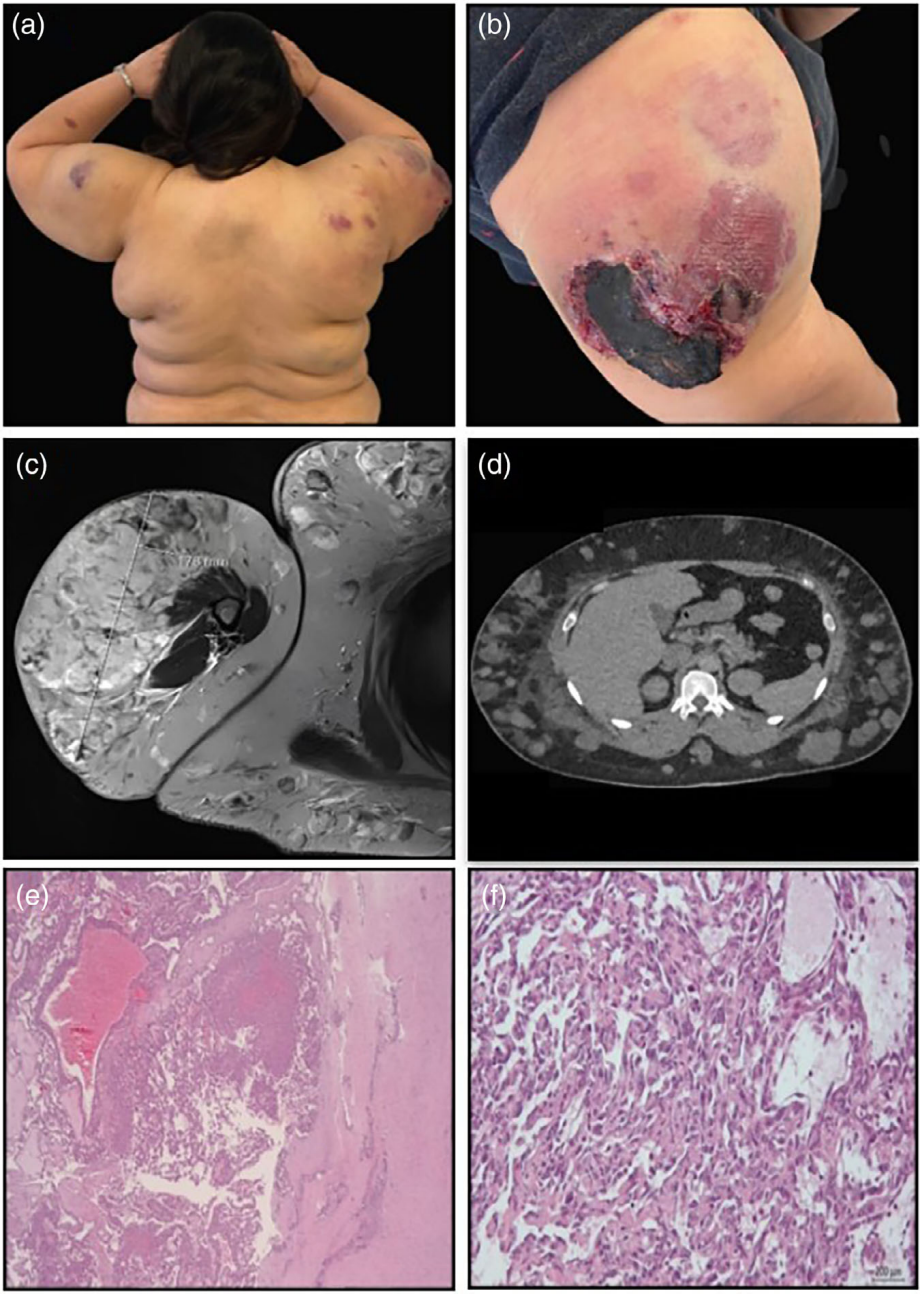
Clinical, radiological, and histopathological presentation. (a) Clinical photograph: multiple subcutaneous nodules. (b) Clinical photograph: tumor of the right upper arm. (c) MRI: tumor of the upper arm. (d) CT: subcutaneous nodules of the trunk. (e) Histopathology: × 25. (f) Histopathology: × 200.

Magnetic resonance imaging (MRI) displayed a mixed vascular mass measuring approximately 15 × 10 × 18 cm on the right upper arm, as well as multiple disseminated subcutaneous masses throughout the entire integument (Figure 1c). Using contrast‐enhanced computed tomography (CT), the posterior circumflex humeral artery could be identified as the predominantly supplying vessel (Figure 1d). Blood tests revealed thrombocytopenia, microcytic anemia with a hemoglobin level of 6.8 g/dl, decreased fibrinogen and Quick values, and elevated D‐dimers. Based on these findings, the patient fulfilled the diagnostic criteria for disseminated intravascular coagulation (DIC). Genetic testing of lymphocytes from EDTA blood and of cultured fibroblasts obtained from a skin biopsy adjacent to one of the subcutaneous masses did not reveal any gene variants associated with known genetic syndromes involving vascular malformations. Histological examination showed numerous slit‐like vascular spaces throughout the entire dermis with slightly enlarged endothelial cells (Figure 1e). The subcutaneous nodules consisted of densely packed and irregularly shaped capillaries with a higher degree of pleomorphism, cytologic atypia and some mitoses (Figure 1f). Solid areas composed of spindle cells resembling Kaposi sarcoma were observed. The surrounding lymphocytic infiltrate contained only few plasma cells. Immunohistochemical analysis showed strong positivity for CD31, CD34, and ERG in vascular endothelial cells of the dermis and subcutis, with a surrounding SMA‐positive pericyte layer. The proliferation rate in the deeper areas was increased, with up to 30% Ki67‐positive cells, indicating marked angiogenic activity. HHV8 testing was negative. The proliferating vascular structures in the dermis and subcutis were largely negative for podoplanin. After consultation with a reference pathologist, the findings were considered consistent with KHE.

KHE most commonly occurs on the upper extremities, followed by the trunk, with an estimated prevalence of 0.91 per 100,000 individuals.^5^ Although occasional familial clustering has been described, no genetic cause has been confirmed to date. KHE predominantly affects children and adolescents, although isolated cases have been reported in adults.^2^, ^3^ Given its rarity in this age group, there are no established treatment protocols.

Initial management in our patient focused on particle embolization of the primary feeding vessels of the upper arm tumor. As demonstrated by Zhou et al., this approach is effective in improving the Kasabach‐Merritt phenomenon, particularly in cases with extensive lesions in which primary excision is not feasible due to the high risk of bleeding.^6^ However, despite our extensive efforts, the bleeding persisted. Therefore, a novel therapeutic approach, bleomycin electrosclerotherapy (BEST), was attempted one week later. This method involves administration of bleomycin followed by multiple short electrical impulses delivered through fine needles. This process temporarily increases the permeability of cell membranes to the sclerosing agent bleomycin, thereby enhancing its intracellular concentration in the target tissue. Owing to its site selectivity and the lower total body dose required, this approach markedly reduces systemic side effects.^7^ Under general anesthesia, 30 mg of bleomycin – calculated according to body surface area and dissolved in 100 mL of NaCl – was first injected into the tumor of the right upper arm, ensuring even distribution of the solution throughout the area. Subsequently, 57 electrical impulses with an intensity exceeding 14.5 amperes were delivered into the tumor tissue using a hexagonal electrode. A compression bandage was then applied. As the first systemic therapy approach, the patient was started on prednisone 60 mg/day (1 mg/kg body weight), vincristine 2 mg/day, and lenalidomide 10 mg/day.^8^ However, despite the month‐long treatment, the disseminated subcutaneous KHE of the upper body continued to progress, prompting a shift in the therapeutic approach to sirolimus 3 mg/day. This change led to an improvement in DIC, which subsequently enabled excision of the large tumor on the upper arm.^6^, ^9^ A 6‐month follow‐up CT staging showed moderate regression of the remaining smaller KHE lesions of the integument.

This case highlights the diagnostic and therapeutic challenges of managing KMP in adults and proposes a potential novel local treatment approach using BEST. Specifically, for bleeding control and the initial management of extensive or hemorrhagic lesions that cannot be primarily excised, this approach may serve as a valuable complement to systemic therapy.

## CONFLICT OF INTEREST STATEMENT

None.
